# A Study of Public Attitudes toward Shanghai’s Image under the Influence of COVID-19: Evidence from Comments on Sina Weibo

**DOI:** 10.3390/ijerph20032297

**Published:** 2023-01-27

**Authors:** Yanlong Guo, Lan Zu, Denghang Chen, Han Zhang

**Affiliations:** 1 Social Innovation Design Research Centre, Anhui University, Hefei 203106, China; 2 Anhui Institute of Contemporary Studies, Anhui Academy of Social Sciences, Hefei 203106, China; 3 Department of Science and Technology Communication, University of Science and Technology of China, Hefei 203106, China; 4 College of Environmental Science and Engineering, Ocean University of China, Qingdao 266000, China

**Keywords:** city image, COVID-19 epidemic, social media, LDA, sentiment analysis

## Abstract

With the advent of the Internet era, Chinese users tend to choose to express their opinions on social media platforms represented by Sina Weibo. The changes in people’s emotions toward cities from the microblogging texts can reflect the image of cities presented on mainstream social media, and thus target a good image of cities. In this paper, we collected microblog data containing “Shanghai” from 1 January 2019 to 1 September 2022 by Python technology, and we used three methods: Term Frequency-Inverse Document Frequency keyword statistics, Latent Dirichlet Allocation theme model construction, and sentiment analysis by Zhiwang Sentiment Dictionary. We also explore the impact of the COVID-19 epidemic on Shanghai’s urban image in the context of the “Shanghai Territorial Static Management”, an important public opinion topic during the COVID-19 epidemic. The results of the study show that the “Shanghai-wide static management” of COVID-19 epidemic has significantly reduced the public’s perception of Shanghai and negatively affected the city’s image. By analyzing the data results, we summarize the basic characteristics of Shanghai’s city image and provide strategies for communicating Shanghai’s city image in the post-epidemic era.

## 1. Introduction

City image is the public’s overall impression and evaluation of the city’s material elements such as economic resources, infrastructure, and other non-material elements such as history and culture. It not only reflects the city’s historical accumulation and development status, but also contains the prospect of the city’s future development potential. The perception, feeling, and opinion of the public on the city image are of great significance to the city management, city planning, city culture perception, and the development of tourism resources. At the same time, the city image also reacts to the residents’ living happiness. A good urban image of the city where people live can enhance residents’ sense of self-identity and reduce their psychological pressure in daily life, as well as play a positive role in maintaining residents’ mental health [1,2,3].

As the Internet enters the era of Web2.0, social media platforms represented by Facebook, Weibo, Zhihu, and Twitter have significantly broken through the limitations of traditional media in information production and dissemination with their distinctive characteristics of openness, timeliness, interactivity, and connectivity [4]. According to the Digital 2021: The World at a Glance report, social media users grew by more than 400 million in the last 12 months, reaching 4.55 billion by October 2021. User growth has slowed slightly over the past three months, but the global total continues to grow at an even higher rate, with more than 1 million new users per day [5]. Based on such a large and diverse audience group, the application of social media means huge communication potential and opportunity, creating a brand-new space for the construction of urban image.

Currently, group discourse in social media is increasing, and Sina Weibo has come to be one of the necessary channels for the public to attain real-time data and discuss trending current affairs. As one of the social media systems, Sina Weibo can collect a large number of users’ most real thoughts. Especially, during the epidemic, the attributes of Weibo as a public dialogue platform grew to be significant, turning into the most essential platform for the public to acquire records about the epidemic and to exchange and discuss information. According to Weibo Data Center statistics, 37,000 government micro-blogs and 3000+ media micro-blogs launched 6,076,000 portions of authoritative records about the epidemic in 2020, with a total of 364.7 billion reads and initiated 30,000 stay-at-home announcements of the epidemic, with over three billion viewers [6]. As a public social platform, Weibo is able to gather media, parties, government official micro-blogs, and big vloggers in various fields to interpret events from multiple perspectives. These features make Weibo an important channel for shaping and spreading the city’s image.

In the age of the digital network, people have changed from passive to professional consultation to active access to information through various Internet channels [7]. As people are quarantined at home due to the epidemic, social activities have decreased, and the public tends to choose online social platforms to express their personal views. With the development of social media, major social networking media, represented by Weibo, have become platforms for the public to express their personal views on the epidemic and follow the trend of the global epidemic. Digging into public perceptions of city image since the epidemic on Weibo can help relevant organizations understand the image of cities presented on mainstream social media. However, the existing literature rarely deals with the research of urban image regarding this topic.

The research object of this paper is the urban image of Shanghai and makes use of internet crawler technological knowledge to attain micro-blogs containing “Shanghai” from 1 January 2019 to 1 September 2022. Duplicate comments and noisy data were removed to prevent users from repeatedly posting a large number of the same comments and advertising messages that are not related to the topic. Term Frequency-Inverse Document Frequency keyword statistics and Latent Dirichlet Allocation topic modeling were performed on the processed tweets to interpret the keywords and topics displayed for Shanghai each year. This paper similarly quantifies the sentiment value of Shanghai as a town at each time point: the usage of sentiment evaluation is based entirely on the Zhiwang Sentiment Dictionary and analyzes its sentiment value trends from 1 January 2019 to 1 September 2022. Finally, the city image of Shanghai is derived, and the communication strategy of Shanghai city image after the unexpected event is proposed based on the analysis results. By improving the urban image of Shanghai, Shanghai residents can enhance their sense of self-identity.

## 2. Literature Review

### 2.1. Definition of City Image

“Image” refers to an abstract feeling and perception, a perception of an area, or a combination of people’s opinions, judgments, and emotions [8,9]. Lynch, an American urbanist, first proposed the term “city image” in 1964, which he believed was once an impact shared through a positive range of town residents, including roads, boundaries, areas, nodes, and landmarks [10]. Lynch’s metropolis picture is constrained to the impressions that town residents have of their area. This is limited to the understanding of the city’s physical structure, while later researchers put unique emphasis on the influence of non-residential regulations [11,12,13]. The future improvement expectation of a metropolis is also the reflection of its characteristics and charm [14,15].

With the development of modern network, some scholars have suggested that the construction of city image is a process of external communication, that is, the city image is composed of mass media, individual experiences and environmental factors [16]. In addition, some scholars believe that the media is the fundamental device for metropolis photo communication, and the media completes records about the city, which in turn influences public perception and eventually forms the city image [17,18,19,20,21]. The importance of media communication of city image is reflected in its timeliness and comprehensiveness [22].

City image is not only a kind of social existence, but also a kind of social evaluation, which is subjective. The shaping of urban image is also a process of public perception and re-evaluation of the city [23,24]. Studies have shown that a good city image has a positive effect on the mental health of city residents. The building of city image can not only bring economic benefits to the city, but also improve the residents’ happiness, self-identity, and sense of value [25].

### 2.2. Emergencies

Public emergencies refer to natural disasters, accidents, public health incidents and social security incidents that occur suddenly and cause or may cause serious social harm and need to be dealt with by emergency measures. In recent years, scholars at home and abroad have launched research on information dissemination in public emergencies. Theja Bhavaraju et al. studied the changes of indicators such as the duration of attention and emotion of disaster events on Twitter [26]; Kumar et al. applied the contents of memory model to the analysis of a massive range of tweets, pictures, and videos to identify the content of Twitter information related to emergencies in order to facilitate timely decision-making [27]; Ray et al. used supervised learning to distinguish true information from false information about disaster events as presented in social media to mitigate the negative impact caused by rumors [28]. From the existing research results, there are fairly few studies on the trend of public sentiment fluctuation in predominant public fitness emergencies and the impact on the image of cities, and even fewer research results on the theme of mining and sentiment analysis of micro-blog users using the Latent Dirichlet Allocation theme model.

COVID-19 spread primarily through respiratory droplets and contact, is a new infectious disease that is generally contagious to humans [29]. On January 30, 2020, WHO listed COVID-19 epidemic as a public health emergency of international concern, which has the characteristics of infectivity, suddenness, complexity and persistence. Public health events are serious hazards, not only affecting people’s health, but in serious cases hindering economic development and even threatening social stability. Therefore, analyzing the trend of micro-blog users’ emotional fluctuation is a way to reap the public’s subjective understanding of the city’s image, which helps the relevant parts of the government to target the information dissemination of the epidemic online., eliminate social panic, and increase the public’s resolve to defeat the epidemic.

### 2.3. Study of Term Frequency-Inverse Document Frequency Topic Model

The spread of social and communication technologies online has led to the use of social media by many people and the timely participation of the public in emergency response [30,31]. More and more people are using social networking offerings (e.g., Twitter, Facebook, or Sina Weibo) to share things around them [32]. Rapidly advancing machine learning and text mining technologies make it possible to analyze social media data and understand people’s behavior, reactions, and public opinion in emergency situations [33,34]. Politis analyzed tweets before (2019) and during (2020) the outbreak of the epidemic through the Latent Dirichlet Allocation model to study the effect of the pandemic on customers of London’s public transport system [35]. Dahal used the Latent Dirichlet Allocation model to extract topics related to climate change from Twitter data and calculate the sentiment index [36]. Ye divided the dengue-related micro-blog into five themes to study the relationship between micro-blog and dengue fever [37]. Ko et al. analyzed the major data of news reports, modeled the topic based on the Latent Dirichlet Allocation algorithm, and discussed the influence of COVID-19 on education policy and people’s lives in the post-epidemic era [38]. Zong has analyzed the content and timing of Weibo posts related to the chemical plant explosion in Tianjin, China [39]. Peng et al. used Term Frequency-Inverse Document Frequency and Latent Dirichlet Allocation to mine different geographical hot-spots and different individuals or groups in the city through the data of Weibo in Beijing in 2016 [40]. This paper aims to identify public perceptions of Shanghai city image from social media during COVID-19 epidemic, to identify the topics of micro-blog posts related to Shanghai during COVID-19 epidemic, and to reveal changes in public sentiment perceptions of Shanghai city image during this period.

### 2.4. Sentiment Analysis Study

Sentiment analysis uses text mining techniques to identify and extract information for project evaluation and monitoring to aid decision-making [41]. Lexicons and machine learning are frequent strategies for sentiment classification [42]. One study grouped the tweet matters of Twitter customers to gain their opinion on COVID-19 vaccination program [43]. Ahmed et al. collected tweet data over time to study trends and emotional dynamics of the COVID-19 outbreak [44]. Ridwan et al. used Twitter statistics to apprehend the sentiment of public on COVID-19 outbreak in Singapore [45]. However, thematic modeling, extraction, or sentiment analysis regarding public perceptions of city images during the novel coronavirus outbreak remains to be studied. Jim Samuel et al. analyzed public sentiment related to the outbreak using coronavirus tweet text data [46]. Gencoglu et al. developed a causal model using social media data related to the novel coronavirus outbreak to study the spread of misinformation about the coronavirus. A causal relationship between Twitter and public sentiment during the epidemic was determined [47].

The main method currently used for sentiment mining is to use sentiment dictionaries, which match sentiment words to texts, aggregate sentiment words for scoring, and finally interpret the sentiment tendency of texts. The sentiment evaluation approach used in this paper is the Zhiwang sentiment dictionary, which affords a complete set of 12 files, divided into English and Chinese. Among them, the Chinese sentiment dictionary consists of evaluation, sentiment, assertion, and degree (positive and negative) of sentiment texts. In this paper, the evaluation and emotion words are integrated and used as emotion dictionaries. The degree words contained in the degree word list are divided into six emotion degree dictionaries according to the rank distinction: highest–very–more–slightly–insufficiently–too much.

## 3. Model Methodology

### 3.1. Term Frequency-Inverse Document Frequency (TF-IDF) Keyword Statistics

The TF-IDF method is divided into two parts: word frequency (TF) and inverse document frequency (IDF). TF identifies the frequency of words. The calculation method is shown as Formula (1):

(1) $${\mathrm{tf}}_{i,j}=\frac{n_{i,j}}{\sum_{k}n_{k,j}}$$

In the formula, the place i is a one-of-a-kind word, j denotes a diverse tweet, and $n_{i,j}$ is the variety of occurrences of phrase i in tweet j, and $\underset{k}{\sum }n_{k,j}$ denotes the wide variety of occurrences of all phrases in tweet j.

IDF is used to identify the importance of phrases. It can increase the importance of rare phrases and reduce the importance of common phrases, as shown in Formula (2):

(2) $${\mathrm{idf}}_{i}=\log\frac{\left|D\right|}{\left|j:t_{i}\in d_{j}\right|+1}$$

where $\left|D\right|$ is the whole quantity of tweets, and $|j:t_{i}\in d_{j}$| indicates the wide variety of tweets containing the phrase $t_{i}$. If all tweets do not comprise the $t_{i}$ the word, this will result in $\left|j:t_{i}\in d_{j}\right|$ =0. To keep away from a denominator of 0, we choose $j:t_{i}\in d_{j}|+1$.

Finally, multiply the result value of Formula (1) with that of Formula (2) to get the value of TF-IDF, as shown in Formula (3). This means that the larger the TF-IDF value, the more important the phrase:

(3) $$\mathrm{tf}-\mathrm{idf}={\mathrm{tf}}_{i,j}\times {\mathrm{idf}}_{i}$$

### 3.2. Latent Dirichlet Allocation Topic Model

Hot words in the network can be extracted directly by word frequency statistics but cannot identify the semantic correlation behind the phrases. Different phrases may have the same thematic context, such as “the thunder is loud” and “I do not have an umbrella”, each of which reflects the subject matter of “rainy weather”. However, if solely the phrase frequency is counted, it is difficult to get the real theme of the text, because each phrase is not necessarily identified as a high-frequency phrase.

LDA is a model that considers semantic associations between texts. LDA is a three-level Bayesian probabilistic graphical mannequin with three granularities: document, topic, and phrase [48]. The LDA model mines the potential topic information in the document set or corpus and constructs a model using bag-of-words, which constitutes "document-topic distribution" and "topic-word distribution" without considering the order of word occurrence [49]. One or extra subjects represent a document, and every phrase in the file is generated by means of one of the subjects [50].

In order to deal with public sentiment in the context of the epidemic and to grasp the problems brought on by the image of the city, the LDA model can assist in the procedure of text-based evaluation through practicable theme identification and person-clustering. The key to using the LDA is to determine the most appropriate broad topic category. The effectiveness of subject matter origin in the LDA is immediately associated with the variety of attainable themes. Coherence or perplexity [51,52,53,54] are the two most common ways to determine the number of topics. Coherence is special as to whether the semantic affiliation of phrases beneath the matters generated by using the quantitative LDA calculation is tighter, and the formulation for calculating subject matter coherence is proven in (4):

(4) $$\mathrm{Coherence}\left(T\right)=\sum_{\left(v_{i},v_{j}\right)\in T}p\left(v_{i},v_{j}\right)$$

where T is the subject of $v_{i}$ and $v_{j}$ are the phrases inside the topic, and $p\left(v_{i},v_{j}\right)$ represents the scoring function of phrase semantic approximation.

In general, the feature $p\left(v_{i},v_{j}\right)$ is typically used in the UMass algorithm [36], Formula (5):

(5) $$p\left(v_{i},v_{j}\right)=\ln\frac{p\left(v_{i},v_{j}\right)+\varepsilon }{p\left(v_{i}\right)\;p\left(v_{j}\right)}$$

where $p\left(v_{i},v_{j}\right)$ the table shows the word $v_{i}$ and $v_{j}$ are the co-occurrence probabilities of $p(v_{i})$ and $p\left(v_{j}\right)$ show the co-occurrence probabilities of the words $v_{i}$ and $v_{j}$ and the chance of occurrence of the phrase ε is a small constant.

To sum up, this paper selects the evaluation methods of big coherence and small coherence to obtain the number of themes and complete the construction of the LDA.

### 3.3. Sentiment Analysis by Zhiwang Sentiment Dictionary

Emotion analysis extracts the attitude tendency of text, which belongs to the sub-field of natural language processing. We used the informed dictionary for sentiment analysis and achieved a wide range of accurate sounding phrases and complex language rules to generate sentiment values. After identifying sentiment words, the sentiment intensity of phrases is measured, assigning lower scores to degree words that are further away from the sentiment word primarily based on adverbs, negation, punctuation, and conjunctions.

**Figure 1:**
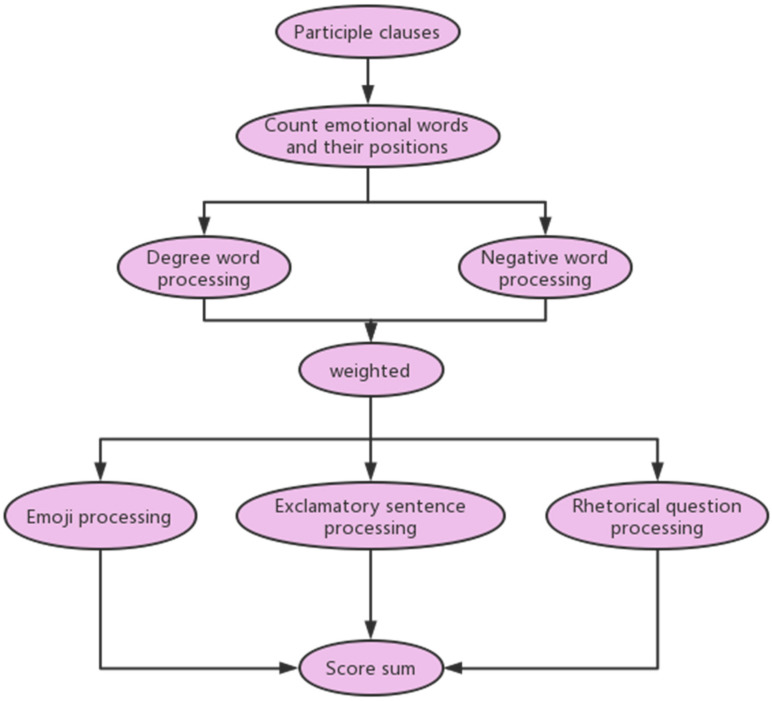
Emotion analysis flow chart.

The number of sentiment phrases in the passage is decided by means of including 1 with the variety of positive phrases and 1 with the variety of negative phrases, and in the process of counting the variety of sentiment phrases, it is additionally vital to decide whether or not there is a degree adverb earlier than the sentiment word, and if so, it is imperative to provide extraordinary weights in accordance to the kind of degree adverb and multiply it by means of the range of sentiment words. If a sentence ends with an exclamation point (!) or a question mark (?), such punctuation marks often indicate the strengthening of emotional reactions, so a certain number of emotional words needs to be increased. Then, the sentiment value (positive words–negative words) of the whole paragraph is calculated, and the sentiment tendency of the paragraph is obtained. Finally, the sentiment value of every paragraph is counted and summed to obtain the complete sentiment value of the textual content. The overall flow block diagram is as follows (Figure 1):

## 4. Data Statistics and Analysis

### 4.1. Data Collection

We used micro-blogging data sources for our research: blog posts containing “Shanghai” from 1 January 2019 to 1 September 2022 were collected pro-grammatically in Python. To obtain a clearer picture of the adjustments in the data, we divided the data into four quarters each year (three months make up a quarter) and collected blog posts with the words “Shanghai” from 1 January 2019 to 1 September 2022. The whole quantity of weblog posts with the phrase “Shanghai” in 11 groups is 100,438; for the purpose of analysis, we will be in the first quarter of 2020 to 2022 in the third quarter respectively. Following are the numbers for A1, A2, A3, A4, B1, B2, B3, B4, C1, C2, and C3 (Table 1):

### 4.2. Data Processing

(1) This study uses web crawler technology to collect all micro-blogs containing “Shanghai” on micro-blogging platforms from 1 January 2019 to 1 September 2022. First, we cleaned the data: the original micro-blog data contained a lot of confusing codes, characters, links and other useless contents. This data needs to be cleaned to obtain a more standardized dataset. The meaningless symbols such as “@, #, [], []”, URL links, and all numbers and foreign languages (including Japanese, Korean, and English) were filtered out to reduce the data noise. Finally, “Shanghai” was excluded because all blog posts contain the word “Shanghai”, which is not practical for the follow-up study.
(2) Splitting and deactivating words was then completed. Micro-blog text is composed of individual words. In this paper, the corpus is divided and deactivated with the help of a pkuseg Chinese word separation toolkit, a new phrase separation kit which greatly improves the accuracy of phrase separation for exclusive domain information. According to the results, pkuseg reduced the word separation error rate by 79.33% and 63.67% on the example datasets (MSRA and CTB8), respectively [55]. After generating a word frequency list, a custom deactivation word list was generated by manual screening, the text was re-filtered, and the pre-processed Shanghai Weibo data were subjected to TF-IDF keyword statistics and subject mining and sentiment analysis using LDA topic models.

### 4.3. Keyword Statistics

We first use the TF-IDF method to calculate the TF-IDF value, and then we sort the keywords. The occurrence times of keywords were obtained by word frequency statistics.

The novel coronavirus outbreak of 2020 swept everywhere, generating topics of discussion around the world. Table 2 shows the top 20 keywords for A1 and A2 in the first and second quarters of 2020. In the first quarter, “statement” and “response” ranked first and second, respectively, indicating that people attached great importance to government statements and responses to questions related to the outbreak at the beginning of the novel coronavirus outbreak. The TF-IDF value of “masks” ranked third, and there was an extreme shortage of resources for masks at the beginning of the outbreak, which became the focus of people’s discussions. Since Wuhan was the first city in China to experience the new epidemic, the keyword “Wuhan” ranked sixth. In addition, “Red Cross”, “hospital”, “epidemic”, “doctor”, and “supplies” entered the list. These five words entered the top 20 keywords in the first quarter of 2020, indicating the high level of attention and discussion about the new Guan epidemic on Weibo during this period.

“Police stations” and “police” were in the fourth and eleventh places respectively, indicating that in addition to medical personnel, police were also involved in handling the epidemic in the face of the outbreak. Positive words such as “support”, “cheer”, “hope”, and “peace” also appear, expressing people’s support for overcoming the epidemic. This indicates that the public sentiment in COVID-19 period was generally positive. In addition, the word “program” appears in the 18th position. In January and March, during the Chinese Spring Festival, the CCTV Spring Festival Gala 2020 added a special program on the fight against the epidemic, focusing on the medical workers and people who were fighting on the front line of the epidemic, conveying the strong confidence and determination of the people. The program was widely praised, and the ratings of the Spring Festival Gala increased significantly compared with previous years.

From the keyword ranking in the second quarter of 2020 (Table 2), “testing” appeared in the first place in the second quarter of 2020, and nucleic acid testing was the first and most effective way to detect the novel coronavirus, which was frequently discussed at the beginning of the epidemic. Among the top 20 keywords, many positive adjectives ranked high, such as “cute”, “good-looking”, “happy”, “good “. Clearly Shanghai’s city image is dominated by means of positive adjectives, many of which are associated with the preliminary victory of China’s epidemic prevention and management. The keywords “cheer”, “spring”, “meaning”, “finally”, “expectation,” “hope,” and “opportunity” all indicate people’s confidence and expectations in overcoming the epidemic. The word “wage” appears in the sixth place, indicating that the economic downturn has affected people’s income sources under the epidemic situation.

In the third quarter of 2020 (Table 3), the epidemic situation improved, and entertainment venues previously closed due to the epidemic were gradually opened in various places, and the top 20 keywords include “movie”, “acting”, “good-looking”, and “not to be seen”, indicating that people’s entertainment lives have begun to become enriched. The variety of positive adjectives accelerated whilst the quantity of epidemic-related discussions lowered extensively in the top 20 key phrases.

In the fourth quarter, “happy” rose to the first place, and the adjectives are still mainly positive, indicating that the image of Shanghai has been improved. The fourth quarter coincided with the handover of the old and new years, and the keywords “New Year”, “New Year’s Eve”, and “fireworks” were added, while “expectation” and “cheer” were added as well. The words “expectation”, “cheer”, “hope” and “hard work”, which express good expectations for the New Year, remained at the top of the keyword rankings. In addition, “Disney” also became a new word in the top 20 keywords in the fourth quarter, and Shanghai, as the only city in China with Disneyland, will also become a topic of interest. Words like “epidemic”, “hospital”, “mask” and other words related to COVID-19 epidemic no longer appear in the top 20 keywords.

Among the top 20 keywords in the first quarter of 2021 (Table 4), “Legal” ranked first, and the effective date of the Civil Code of the People’s Republic of China was 1 January 2021, which was frequently discussed by the public. Positive adjectives such as “good-looking”, “looking forward to” and “happy” remain in the top 20 keywords, while epidemic-related words still do not appear in the top 20 rankings. The words “Weibo”, “Blockbuster”, “Haute Couture” and “Loan” were added to the top 20 keywords related to entertainment news at the time. The term “public opinion” was also added. The word “public opinion” was also in the top 20, which was more about the closure of the city of Wuhan. The positive adjectives in the second quarter remained the same as the previous quarter, with the addition of “red-cooked pork” in the fifth place, which has become a classic dish of Shanghai’s local cuisine and almost an icon of the city. In addition, “live” and “teacher” also appeared in the top 20 keywords. In order to prevent the epidemic from harming education throughout China, elementary school, junior high school, high school, and teachers in universities around the world have been holding live webcast lectures. Online classes have become a new way of teaching in the education industry. The live broadcasting industry also emerged during this period, and live streaming became a new means of online sales.

In the third quarter of 2021 (Table 5), “house” and “house price” were among the top 20 keywords, and Shanghai’s house prices soared in this quarter, becoming the focus of people’s attention. In addition, the discussion of Shanghai on Weibo at this time was still dominated by positive words, showing the positive image of Shanghai. Among the top 20 keywords in the fourth quarter, “happy”, “good”, “lovely”, “good”, “congratulations” and other positive words are still in the top 20. In addition, “Disney”, “Halloween”, “New Year”, “fireworks” and other words with a festive and cheerful atmosphere were ranked in the top 20. At the same time, “landing” and “smooth” also became new words in the fourth quarter, corresponding to the second half of the postgraduate exams and national civil service exams; during the epidemic, people prefer to take public exams to improve themselves. However, “nucleic acid” and “epidemic” reappeared in the top 20 keywords in the fourth quarter, ranking seventh and ninth respectively, indicating the recurrence of the epidemic in Shanghai at the end of 2021.

In the first quarter of 2022 (Table 6), although “happy” still ranked first in the public event of the COVID-19 epidemic in Shanghai, the positive adjectives in the top 20 keywords decreased significantly, with “doctor”, “epidemic“, “vaccine”, “virus”, “quarantine”, “ambulance” These words made it into the top 20 keywords. Locations such as “city”, “neighborhood” and “Pudong” also became new keywords, and Weibo once again became an important social media platform for people to learn about the new situation of the epidemic in Shanghai. In the second quarter, the epidemic continued to fester, with “epidemic” rising to second place and “nucleic acid” rising to 14th place. The number one keyword was “banks”, while “withdrawal”, “depositors”, “material” and “deposit” ranked first. The fourth, fifth, sixth and ninth rankings for “deposit” indicate that the epidemic led to increased economic pressure on Shanghai citizens and the lack of materials became a serious social problem in Shanghai at that time. “State” and “government” are also new words, indicating that people hope the state and government can take effective measures to solve the problem in response to the epidemic in Shanghai. The word “online” reappeared in the top 20 keywords, as schools and enterprises adopted online teaching and online offices in response to the epidemic. “Henan” and “villages and towns” appeared in the 11th and 12th places, and there was a recurrence of the epidemic in Henan during the same period.

In the third quarter (Table 7), the epidemic was effectively controlled, and “epidemic” dropped to the 20th place. “Typhoon” became the first keyword: Shanghai was affected by Typhoon Songda, Shanghai Meteorological Bureau launched typhoon level IV emergency response at 10:00 on 29 July 2022, and Shanghai Flood Control Command decided to launch the city’s flood control and typhoon level IV response at 9:40 on 30 July 2022. The fact that “nucleic acid” rose to second place indicates that nucleic acid testing is becoming the norm and an effective way to screen for the novel coronavirus. “Court”, “judge” and “police” are new words added in the third quarter, indicating the interest in civil or criminal cases to be resolved after the outbreak. “The elderly” entered the top 20, ranking 15th. The most affected group of the epidemic is the elderly, who are in poor health, and many of them have difficulty resisting the attack of the novel coronavirus. “Moutai” ranked eighth, indicating that public attention began to shift partially to Moutai’s share price. Meanwhile, “spokesperson”, “brand”, and “global” ranked seventh, ninth, and tenth respectively, indicating that Shanghai, as an international fashion city, has increased public attention on international fashion brands after the epidemic has subsided. Positive words such as “cheer”, “expect”, “hope”, and “good” reappear in the top 20 keywords.

In this article, we counted the top 20 keywords in Weibo posts about Shanghai from January 2020 to September 2022. Positive words such as “happy”, “good”, and “good-looking” appear more often in these three years. The number of positive words in the top 20 declined significantly during the period when the epidemic first broke out in January–June 2021 and during the sudden and serious epidemic in Shanghai in January–June 2022, with “epidemic,” and “virus” appearing as keywords. The number of epidemic-related words such as “nucleic acid” and “hospital” has increased, indicating that Shanghai’s image will be negatively affected in 2022 due to the large-scale epidemic closure event.

### 4.4. Theme Mining

The number of topics is determined by two metrics, perplexity and consistency. In practical applications, adjusting the number of topics only according to perplexity may result in topic extraction results that cannot be interpreted by humans. Therefore, in this paper, we typically refer to the consistency rating to decide the wide variety of topics. Appendix A indicates the consistency of the LDA topic model (Coherence) for finding out the quantity of subjects for every quarter from Q1 2020 to Q3 2022. Higher Coherence scores indicate better-aspect interpretability, meaning the scores are more meaningful, and also more semantically coherent. Based on the Coherence graph in Appendix A, the number of topics for quarters 1–4 in 2020 is determined as 8, 5, 7, and 5, respectively; for quarters 1–4 in 2021 as 9, 6, 8, and 4, respectively; and for quarters 1–3 in 2022 as 4, 7, and 4, respectively (Table 8). Table 9 Table 10 Table 11 Table 12 Table 13 Table 14 Table 15 Table 16 Table 17 Table 18 Table 19 show the outcomes of the LDA theme model for A1-C3.

In the results of the first quarter of 2020 (Table 9), Topic1 reflects the panoramic police documentary “No Small Matter in a Big City—Stories from Police Stations 2019” jointly produced by Shanghai Oriental TV and Beeping, which was given an award in the first quarter of 2020 by the State Administration of Radio and Television (SARFT) for being an innovative program for radio and television. Topic2 focuses more on the U.S. presidential election, with words such as “America” and “government”. Topic3 introduces tourism, mainly consisting of words such as “city”, “tourism” and “Zhejiang”, including “Mickey”, which is related to Shanghai Disneyland. “Topic4, Topic5, and Topic6 are all related to COVID-19 outbreak, with Topic4 focusing on the official response of government departments to COVID-19 outbreak. Topic5 reflects the people of Shanghai welcoming home the medical staff supporting the Wuhan area. Topic6 reflects the discussion of the epidemic and hospitals in Wuhan and Beijing, etc. Topic7 reflects the positive sentiment of the people of Shanghai about the current state of life. Topic8 primarily comprises the comments of Shanghai people on entertainment news.

In the results for the second quarter of 2020 (Table 10), Topic1 revolves around the start of the new semester during the epidemic. People are worried about the movement of people around the country plus the infection of COVID-19, and most universities in China have adopted a policy of delaying the start of school. Topic2 and Topic3 reflect the discussion of economic issues in the Shanghai area after the first wave of the epidemic has stabilized. Topic2 mainly focuses on several aspects of wages, individual business, and housing prices, while Topic3 focuses on contract renewal, income, and air conditioning transportation costs. Topic4 mainly discusses the new high-speed rail lines that will open for operation in 2020, and the continued expansion of the rail network between cities, shortening the travel time between cities. Topic5 primarily discusses the content related to the League of Legends Summer Tournament, with words such as “good*looking”, “cheering”, and “awesome”, indicating that the public’s attention gradually shifted to other aspects after the epidemic improved.

In the results for the third quarter of 2020 (Table 11), Topic1 reflects the rescue of wildlife, including the words “rescue”, “animal”, “protection”, and so on. Topic2, Topic3 and Topic5 reflect people’s discussions on games and entertainment stars, with Topic2 focusing on games, Topic3 on entertainment stars, and Topic5 on movie and entertainment works. Topic4 is a discussion of the problem of illegal registration of web links in the context of the new epidemic, and Topic7 is a discussion of people’s concerns about food, including the discussion of fruit prices, such as “delicious”, “value”, “sunshine rose”, etc. During this period, the epidemic situation in Shanghai was stable, and people’s attention was more focused on daily life and recreation.

In the results of the fourth quarter of 2020 (Table 12), Topic1 reflects people’s expectation of New Year’s Eve party programs, such as “stage”, “New Year’s Eve”, and “expectation”. Topic2 reflects people’s discussions on daily life, and Topic3 mainly reflects people’s discussions on tourist cities, including Shanghai “Disney”, as well as “Guangzhou” and “Beijing”, which are closely related to Shanghai. Topic4 reflects people’s expectations for the New Year, while Topic5 is mainly about the upcoming fashion week to be held in Shanghai in the coming year. At this stage, there are no more topics related to the epidemic, and there is a positive trend in general.

In the results of the first quarter of 2021 (Table 13), Topic1 and Topic2 reflect people’s discussions of daily leisure life, with Topic1 focusing more on real-life activities such as “dressing up” and “punching the clock”, and Topic2 focusing more on the emotional expression of Shanghai people’s spiritual world. Topic3 is closely related to the city and consists of regional words such as “country”, “Chengdu”, and “Guangzhou”. Topic4 is a discussion on the scandalous news of celebrities’ illegal behaviors at that time, and Topic5 is a heated discussion on the Civil Code of China, which will come into impact on 1 January 2021. Topic6 focuses on the widespread discussion of the default of debt by, celebrity parents, which consists of the words “borrowing”, “repaying debts” and “restitution”. Topic7 and Topic9 are mainly comments on entertainment, movies, and celebrities, with Topic7 focusing on people’s expectations for the New Year’s “blockbuster” and comments on celebrities’ style of dress. Topic9 is a discussion of fashion, consisting of words such as “beautiful”, “fashion”, and “design”. Topic8 is a discussion on the legal trial of spreading rumors using the Internet.

In the results of the second quarter of 2021 (Table 14), Topic1 is about expectations for the future consisting of words such as “hope”, “time”, and “first”. Topic2 is mainly about upcoming movies, while Topic3 is about movie launches and celebrities’ schedules, including words such as “meeting”, “Chongqing”, and “Changsha”. Topic4 is about festivals, including “May Day”, “June Day”, and “Disney”, a place to celebrate festivals. Topic5 is about people in Shanghai who work in different cities discussing the issue of ticketing after the Spring Festival, including “Beijing”, “Hangzhou”, and “work”. Topic6 reflects the anticipation of celebrity fans for the live stages hosted by celebrities.

In the Q3 2021 results (Table 15), Topic1 reflects the rise of the video software and live-streaming industries, Topic2 reflects discussions about real estate developers and China’s home buying policy, and Topic3 is closely related to cities, with “Beijing” being the city that people in Shanghai follow closely. Topic4 reflects the discussion of social groups such as “takeaway”, “little brother”, “elderly”, and “women”. Topic5 is closely related to the competition, which was one of the topics of discussion during the Tokyo Olympics. Topic7 reflects people’s discussions of old brands in the past, and Topic8 shows the repeated discussions of the epidemic on Weibo, expressing people’s gratitude to the hard-working medical staff. During the period from A3 to B3, the epidemic situation in Shanghai is stable, and the public’s topics are focused on daily life, entertainment gossip, national policies, and the economic situation, and the discussion of the epidemic is significantly reduced.

In the results for the fourth quarter of 2021 (Table 16), Topic1 reflects the increase in nucleic acid testing at amusement parks such as Disney during Halloween in response to the recurrence of the epidemic, and Topic2 reflects the anticipation of the Winter Olympics in China. Topic4 is related to the happiness index of Chinese cities, including the words “Beijing”, “Chengdu”, and “happiness”.

In the results for the first quarter of 2022 (Table 17), Topic1 reflects the seriousness of the COVID-19 epidemic in Shanghai, where a large number of people needed to undergo nucleic acid testing, expressing people’s sympathy for the hard-working medical staff. Topic3 reflects the severity of the epidemic in Shanghai, with the words “doctor” and “vaccine” being related to the epidemic, and “community” being related to the epidemic. The words “neighborhood” and “ambulance” are reflections of the epidemic situation in Shanghai, as ambulances will go to the neighborhoods where the COVID-19-positive patients live and take away the positive patients and their close contacts for isolation. Topic 4 reflects the expectations of the people of Shanghai for the “unsealing” and “clearing” of the epidemic as the New Year approaches. Most of the topics in this period revolve around the epidemic, mainly due to the emergence of socially asymptomatic cases in Shanghai in February 2022, the sporadic closure in early March, the continuous deterioration in mid-March, and the sharp decline in late March, when Shanghai started “local static management”.

Since April 4, Shanghai has entered into a situation of “territory-wide static management”. In the results for the second quarter of 2022 (Table 18), Topic1 reflects the impact of the shutdown on the real economy due to the epidemic in Shanghai, and the major topics of discussion are “banks”, “deposits”, and “depositors”. Topic2 reflects Shanghai people’s comparison of wages and economy between the northern and southern cities, and Topic3 and Topic4 show the management of epidemic prevention and control by the street community, with “epidemic”, “neighborhood committee”, and “government”. Topic5 reflects the material support sent to the people of Shanghai by various governments and companies. Topic6 reflects the economic problems encountered by the people of Shanghai during the epidemic, such as the people’s reduced income and lower consumption levels due to the epidemic, and even some unscrupulous enterprises taking advantage of the opportunity to make national disaster money and harm people’s hard-earned money, showing the negative emotions during the epidemic. Words such as “clear”, “data”, and “go to work” are evident.

By the third quarter of 2022, the epidemic situation in Shanghai gradually improved, people started to resume work and production, and their lives returned to normal. In the results of the third quarter of 2022 (Table 19), Topic1 reflects the landing of Typhoon No. 12 “Meihua” in Fengxian, Shanghai in 2022, including the words “typhoon”, “time” and “place”. Topic2 is related to cities, and the regions that Shanghai people pay close attention to are “America” and “Hangzhou”. Topic3 and Topic4 reflect the court’s handling of online rumors during the epidemic after the epidemic had subsided in Shanghai. With the serious epidemic and the large area closure in Shanghai, citizens in a state of panic and anxiety tend to express their emotional attitudes through social network platforms, and some people use the network platform as a channel to vent their anger, resulting in the instantaneous emergence of a large amount of true and false public opinion information and geometric fission, forming a group polarization phenomenon. This can even lead to secondary public opinion and offline mass incidents, posing a serious threat to social harmony and stability. Topics include “rumor”, “court”, “disinformation”, “network”, “epidemic”,etc.

### 4.5. Emotional Analysis

**Figure 2:**
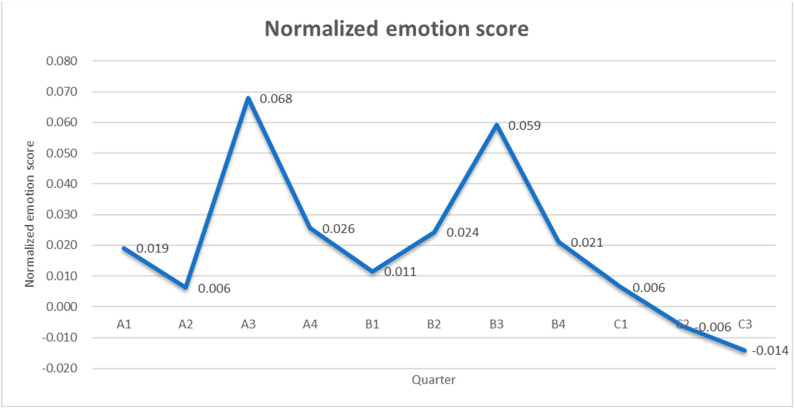
Statistical chart of changes in sentiment values of A1−C3.

Keywords in statistics, such as “cute”, “happy”, “good”, “happy”, “good-looking”, “healthy”, etc., assist us in recognizing people’s emotional perception of Shanghai, because these adjectives express mostly positive emotional tendencies. In this paper, we also quantified the emotional values of the town of Shanghai at a range of time points by using the Knowing Sentiment Analysis method (Table 20). Figure 2 depicts the trend of sentiment values from A1 to C3, and finds that the sentiment values in A2, B1, and C1 are all below 0.02. A2 coincides with the global outbreak of COVID-19, and Shanghai’s city image, as the second largest city in China, is thus affected. Sentiment values for Shanghai were negative for the first time in three years in C2 and C3, with a negative sentiment bias. The sentiment value is even lower in the third quarter of 2022. The public’s sentiment perception of Shanghai was significantly reduced after the “Shanghai-wide static management” event, and the city’s image was seriously affected. People’s perception of the town is carefully related to predominant emergencies.

Compared to an average of 11 quarters, the sentiment values of A1, A2, B1, C1, C2, and C3 are below the mean value, and the sentiment values of C2 and C3 are under 0, displaying a negative sentiment tendency. The sentiment values of A4, B2, and B4 are slightly higher than the average sentiment values and are relatively stable.

Based on the data of keywords, it can be seen that there are many factors affecting public sentiment. For example, major festivals will also affect the public’s attitude toward speech on Sina Weibo. Therefore, we did a cross-sectional analysis of public sentiment in the same quarter over three years of the epidemic. First of all, A2’s emotional value was 0.006, and A3’s emotional value was 0.068, which were the two quarters with the lowest and highest emotional value in 2020, respectively. From the perspective of the epidemic development at that time, A2 was the peak of the epidemic in China. A3 was significantly improved, and life returned to normal, so the emotional value increased. The emotional value of B2 and B3 was 0.024 and 0.059, respectively, showing an upward trend with the improvement of the epidemic. However, the emotional values of C2 and C3 were −0.006 and −0.014, respectively, which showed a downward trend, corresponding to the epidemic situation in Shanghai at that time from local containment to large-scale containment. It was concluded that this was not a normal epidemic situation. Secondly, A3 and B3 are the peak emotional values in 2020 and 2021, respectively, while in the same quarter of 2022 (C3), they are the lowest emotional values in the past three years, indicating that this is not a normal epidemic situation, but the result of the outbreak in Shanghai. Thirdly, in 2020 and 2021, the public’s emotional value was always positive until the severe outbreak in Shanghai (i.e., C2 and C3 periods), when the public’s emotional value fell to negative value, which also means there was an impact of the outbreak on the public, so that the public’s perception of the image of Shanghai will be affected.

## 5. Discussion

### 5.1. Analysis of Shanghai City Image Characteristics

First of all, Shanghai is a city with active economic development. It is located at the junction of Jiangsu and Zhejiang provinces, the mouth of the Yangtze River, one of the water and land transportation hubs in China, the starting and ending points of the Beijing-Shanghai and Shanghai-Hangzhou railroads, and an important aviation center and one of the three major international airports in China. Shanghai’s unique geographical advantages have created prosperity in shipping and trade, and its economic development has also received widespread attention, especially during the epidemic. The massive closure and shutdown of production caused serious losses to the offline real economy, with rising house prices and decreasing income, which brought great economic pressure to the public, and the keywords and LDA models repeatedly showed “house prices “, “economy”, “deposit”, “wage”, “price”, “consumption”, and so on. This shows that micro-blog users cannot discuss Shanghai without mentioning economic issues.

Second, Shanghai is closely connected with other cities at home and abroad. Based on Shanghai’s superior geographical location, its access to other cities is very convenient, and Shanghai is an important port for China’s foreign exchanges. “Nanjing”, “Chengdu”, “Guangzhou”, “Beijing”, and other city terms appear in high frequency words many times. Words such as “America”, “global”, and “world” appear in the keywords and LDA theme models, indicating that people travel between these cities and Shanghai with high frequency.

Thirdly, Shanghai is an important source of fashion culture and fashion industry in China. Shanghai not only has a complete fashion industry chain, but also is an important market for Chinese fashion consumers. “Shanghai International Fashion & Apparel Expo”, “Shanghai International Fashion Culture Festival”, and other large-scale expositions, as well as Shanghai fashion landmarks such as Tianzifang, 1933 Old Square, M50 and SVA Crossing complement each other, forming a closed loop of design, production, dissemination, and consumption. The words “fashion”, “design”, “exhibition”, “clothes”, “embroidery”, and other words became key words.

Fourth, Shanghai presents an image of openness, innovation, and tolerance. Shanghai is home to a young and energetic community, which allows new and interesting behavioral activities to flourish here. On the one hand, online activities such as “live streaming”, “likes”, and “videos” became key words as people were isolated at home due to the epidemic control. On the other hand, during the stable period of the epidemic, offline activities such as “hitting”, “wearing”, “photo”, “movie “, “movie”, “screening”, and other activities appeared in Weibo many times. This shows that Shanghai’s attitude toward innovative activities is more accepting and inclusive.

Finally, the analysis revealed that the image of the city is vulnerable to the impact of unexpected events. Starting from the sudden outbreak of the COVID-19 epidemic in 2020, people’s sentiment values have significantly decreased whenever the epidemic is serious. The sudden and serious epidemic in Shanghai in the first half of the year caused people’s sentiment perception of Shanghai to reach negative values. The words “epidemic”, “nucleic acid”, “supplies”, “quarantine”, “sealing”, and “city closure” indicate the difficult living conditions of Shanghai people at the moment of the epidemic, while the words “hard-earned money”, “wages”, and “prices”, and “blood money” reflect the influence of the epidemic on Shanghai’s economy, and the words “rumor”, “rumors”, and “court” reflect the impact of on-line public opinion and legal issues on Shanghai’s urban image. 

### 5.2. Shanghai City Image Communication Strategy

First of all, among the adjectives in the keywords, words such as “beautiful” and “happy”, which are always associated with urban tourism, are often associated with positive content, making people have a positive feeling about Shanghai. Therefore, in the post-epidemic era, Shanghai can make use of existing resources (such as Disney) combined with Shanghai’s local culture (such as red culture) to promote the city image and improve the public’s attitude toward Shanghai through tourism resources. It is worth noting that holding major national events in Shanghai is conducive to shaping the image of Shanghai. Themed activities such as internationally renowned exhibitions, fashion events and international fashion forums can be relied on to combine Shanghai’s unique cultural temperament with multiple fashion elements. These endeavors can cultivate a city’s intellectual property with lasting charm and influence.

Second, we should focus on improving the image of Shanghai in terms of culture, entertainment, and appearance. In order to improve the public’s negative impression of Shanghai caused by the outbreak of the epidemic, it is necessary to re-explore the value of Shanghai culture, symbolize the representative elements, and strengthen the effect of Shanghai city brand-building from the perspective of visual aesthetics and visual perception. For example, the concepts and symbols of Shanghai time-honored fashion brands, fashion landmarks, and Shanghai style cheongsam culture can be story-shaped and visualized in the communication process to enhance their folk and brand attributes, so as to identify with Shanghai cultural values. From the results of keywords, statistics and LDA thematic model, it can be seen that these rich and colorful cultural resources are far from being fully explored and displayed, and there are few relevant discussions. Chinese media can create sub-accounts with different themes according to audience interests, make full use of pictures and videos to display more intuitive content, tap the photographable resources of Shanghai culture, and spread the image of Shanghai through digital media, so as to improve the public’s emotional perception of Shanghai.

Thirdly, keyword statistics show that the public’s attention to Shanghai occurs mostly at the abstract level (economic, political, and social problems, etc.), whilst ignoring its special sense as a physical space. We should not only pay attention to the virtual image presented on the network, but also improve the city’s physical image construction. Continuing to enhance and create humanistic landmarks in the town, enriching residents’ cultural and recreational life, improving transportation, roads, and ecological environment, and gaining immersive experiences through visible and auditory sensory perceptions can assist humans in structuring a deep and complex image of the metropolis [56].

Finally, attention should be paid to the identification of network opinion leaders and public opinion guidance. In the case of an emergency epidemic, the role of network opinion leaders should be paid attention to. The LDA model proposed in this paper can not only show the objective factual information of Internet users about the development of the epidemic, but also the subjective comments and feelings of Internet users. Especially in the period of epidemic control, the demand of network users for information related to the COVID-19 epidemic has increased rapidly with the passage of time. Since some topics can reflect topics, events, sub-topics, or even derivative topics, the determination of community opinion leaders for each part plays an important role in guiding public opinion toward and conducting more effective public opinion supervision during the epidemic. Therefore, the media, the government, and other institutions can try their best to meet the information topic needs of these opinion fields, so that opinion leaders can maximize their positive energy guidance and public opinion channeling in the network topic community.

Meanwhile, during the period of public opinion outbreak and predominant discussion, media, government, and relevant public opinion supervision departments can recommend diversified thematic information to opinion leaders through appropriate topic selection, and introduce other topics as important as the topic information of the COVID-19 epidemic. Official media and relevant institutions should actively cooperate with non-official media by highlighting a variety of topics to influence the focus of public attention and awareness of the environment and engaging in continuous data tracking analysis. We must avoid network users too concentrated on a class of epidemic information, resulting in information overload, information burnout and negative impact, triggering secondary public opinion events [57].

## 6. Conclusions

This paper collects micro-blogs about “Shanghai” on Weibo from January 2020 to September 2022 through net crawler technology, and analyzes the introduced town image of Shanghai via TF-IDF key-word statistics, LDA subject mining, and sentiment evaluation of Zhiwang. Based on the analysis results, this paper proposes communication strategies to improve the public’s negative perception of the city and promote the city image after public emergencies. According to the research and analysis, the image of Shanghai in micro-blogs shows the following characteristics: first, Shanghai is a city with active economic development. Second, Shanghai has more connections with other cities at home and abroad. Third, Shanghai’s fashion culture and fashion industry are relatively developed. Fourth, Shanghai presents an open, innovative, and inclusive image. Fifth, the public’s perception of Shanghai is susceptible to emergencies.

Based on the analysis of the city image and characteristics of Shanghai, corresponding strategies are further proposed. First, after the epidemic situation is alleviated, the city tourism should be promoted by combining the existing tourism resources with the local culture of Shanghai. Second, we should deeply explore the value of Shanghai culture, strengthen the effect of Shanghai brand-building, and improve the public’s negative impression of Shanghai due to the outbreak of COVID-19. Thirdly, attached importance to the identification of online opinion leaders and the guidance of public opinion is needed. In the post-epidemic era, the city image of Shanghai will be guided to the direction of positive energy while channeling public opinion.

Our research expands on the research of urban image-related fields, with major contributions in the following three aspects. First, in the aspect of methodology, this paper further mined the data about Shanghai city image in Weibo and used TF-IDF method to extract keywords. Thematic analysis is conducted by LDA model and user sentiment is analyzed by Zhiwang sentiment dictionary. The aggregate of the three strategies provides a complete evaluation of micro-blog users’ perceptions of Shanghai city image and forms a text mining framework for social media platforms used in city image investigation. Second, on the theoretical side, the study of unexpected public events and city image is extended, emphasizing that unexpected public events can have a negative impact on the city image; therefore, a communication strategy for Shanghai’s city image is proposed. Third, from the perspective of practical application, Shanghai is in the period of urban construction and recovery after the epidemic. This study provides powerful data support and communication strategies for Shanghai to rebuild a good city image, and thus it helps to improve the self-identity of Shanghai citizens [1,2,3].

However, it is also important to note that the current research has certain limitations. First of all, the groups covered in this article are limited. Weibo users do not represent the total population in Shanghai. Future studies need to improve data integrity. Nearly 80% of Sina Weibo’s user base is made up of people under 30, and more than half of its users have a bachelor’s degree or above. As a result, our data may miss some populations. In the future, we will analyze more social media data and improve user data. Second, the findings in this paper, broadly speaking, make use of textual content data, and the unique record kinds can be similarly improved in the future to consist of images, videos, and audios. In addition, more than one fact can be studied from a couple of perspectives. Third, the scope of this paper is restricted to digital participation. This paper excludes citizens’ offline discussions about Shanghai from its research. Future research has to focus on how offline participation can better help policymakers’ decisions. Moreover, mining data from social media and e-participation structures can facilitate offline engagement activities. This ecosystem view of offline and on-line engagement to enhance the image of cities deserves similar exploration. Fourth, further analysis of the available negative comments has not been conducted. Subsequent research work can analyze negative remark content material identification, negative remark content material clustering, and negative remark conduct formation, and the effects can supply assistance to authorities’ departments and related organizations to develop city image communication strategies.

## Figures and Tables

**Table 1:** Total number of Weibo tweets, January 2019 to September 2022.

Year	Quarterly	Number	Number of Comments
2020	1	A1	8518
	2	A2	6838
	3	A3	4186
	4	A4	8682
2021	1	B1	15074
	2	B2	12500
	3	B3	7855
	4	B4	8499
2022	1	C1	11960
	2	C2	7673
	3	C3	8653

**Table 2:** Top 20 ranked keywords for A1 and A2.

No.	Keywords	TF-IDF Value	Occurrences	No.	Keywords	TF-IDF Value	Occurrences
1	Take a stand	0.0575	1470	1	Testing	0.0363	264
2	Reply	0.0566	1420	2	Lovely	0.0293	195
3	Mouthpiece	0.0338	557	3	Go for it!	0.0256	162
4	Police Station	0.0252	356	4	Good to see	0.0244	152
5	Support	0.0234	321	5	Happy	0.0232	142
6	Wuhan	0.0188	235	6	Wages	0.0202	118
7	Go for it!	0.0184	229	7	Spring	0.0201	117
8	User	0.0174	212	8	Meaning	0.0190	109
9	Hard work	0.0168	201	9	Well	0.0180	101
10	China	0.0168	201	10	Finally	0.0178	100
11	Police	0.0158	186	11	Expectations	0.0169	93
12	Hope	0.0151	174	12	The Summer Race	0.0163	89
13	Red Cross	0.0150	173	13	Start of school	0.0160	87
14	Hospital	0.0146	167	14	China	0.0156	84
15	Peaceful	0.0142	160	15	Infection	0.0143	75
16	People	0.0135	150	16	Hope	0.0141	74
17	Epidemic	0.0131	144	17	Quite a while	0.0141	74
18	Programs	0.0131	144	18	Local	0.0137	71
19	Doctors	0.0121	130	19	Opportunities	0.0137	71
20	Supplies	0.0120	128	20	Thank you	0.0135	70

**Table 3:** Top 20 keywords in A3 and A4.

No.	Keywords	TF-IDF Value	Occurrences	No.	Keywords	TF-IDF Value	Occurrences
1	Expectations	0.0585	257	1	Happy	0.0311	341
2	Congratulations	0.0460	179	2	Expectations	0.0305	333
3	Go for it!	0.0345	119	3	New Year	0.0246	245
4	Juvenile	0.0322	108	4	Crossover	0.0229	222
5	See you soon	0.0313	104	5	Go for it!	0.0208	195
6	Itinerary	0.0298	97	6	Hard work	0.0195	178
7	Movies	0.0289	93	7	Hope	0.0177	157
8	Future	0.0286	92	8	Link	0.0164	142
9	Acting	0.0253	78	9	Disney	0.0162	139
10	Happy	0.0246	75	10	Good to see	0.0155	132
11	Promising	0.0236	71	11	Well	0.0155	131
12	Well	0.0228	68	12	Effort	0.0146	122
13	Rescue	0.0226	67	13	Sprite	0.0140	115
14	Birthday	0.0221	65	14	Next year	0.0139	114
15	Hope	0.0205	59	15	Feelings	0.0138	113
16	Good to see	0.0200	57	16	Web	0.0134	109
17	See you soon	0.0189	53	17	Fireworks	0.0128	103
18	Excellent	0.0183	51	18	Happy	0.0127	101
19	Child	0.0172	47	19	Not bad	0.0122	96
20	Focus	0.0169	46	20	Guardian TV	0.0119	93

**Table 4:** Top 20 ranked keywords for B1 and B2.

No.	Keywords	TF-IDF Value	Occurrences	No.	Keywords	TF-IDF Value	Occurrences
1	Legal	0.0374	975	1	Expectations	0.0315	393
2	Weibo	0.0217	439	2	Happy	0.0302	369
3	Good to see	0.0217	437	3	Well	0.0207	221
4	Fairness	0.0202	396	4	Good to see	0.0199	210
5	Blockbuster	0.0173	321	5	Braised Pork	0.0198	209
6	Expectations	0.0150	266	6	Not bad	0.0187	193
7	Opinion	0.0144	252	7	Movies	0.0187	193
8	Well	0.0143	250	8	Smile	0.0184	189
9	Child	0.0143	248	9	Lovely	0.0176	178
10	Fairness	0.0138	237	10	May Day	0.0169	169
11	Lovely	0.0127	213	11	Child	0.0152	147
12	Hope	0.0127	213	12	First	0.0143	137
13	Happy	0.0122	203	13	Hope	0.0137	129
14	Life	0.0118	194	14	Go for it!	0.0134	126
16	Thanks	0.0104	165	16	Hard work	0.0119	108
17	Loan	0.0100	156	17	Super talk	0.0118	107
18	Haute Couture	0.0099	155	18	Live Streaming	0.0118	107
19	Country	0.0099	155	19	China	0.0117	106
20	Go for it!	0.0097	151	20	Teacher	0.0115	103

**Table 5:** Top 20 ranked keywords for B3 and B4.

No.	Keywords	TF-IDF Value	Occurrences	No.	Keywords	TF-IDF Value	Occurrences
1	Child	0.0340	331	1	Disney	0.0392	374
2	Lovely	0.0274	244	2	Happy	0.0304	259
3	Happy	0.0204	162	3	Hope	0.0288	240
4	Feelings	0.0202	160	4	well	0.0239	186
5	Not bad	0.0193	151	5	Feelings	0.0219	166
6	House	0.0181	138	6	Lovely	0.0217	164
7	Marriage	0.0180	137	7	Nucleic acid	0.0212	159
8	Takeaway	0.0176	133	8	Good to see	0.0212	159
9	Good to see	0.0172	129	9	Epidemic	0.0202	149
10	Life	0.0155	113	10	Not bad	0.0194	141
11	Expectations	0.0150	108	11	Halloween	0.0170	118
12	House price	0.0146	104	12	New Year	0.0170	118
13	hard work	0.0133	92	13	Go for it!	0.0164	113
14	Happy	0.0130	90	14	Fireworks	0.0135	88
15	Awesome	0.0126	86	15	China	0.0133	86
16	Hope	0.0125	85	16	Smooth	0.0130	84
17	well	0.0118	79	17	Thank you	0.0130	84
18	Envy	0.0114	76	18	Congratulations	0.0125	80
19	Air Conditioning	0.0114	76	19	Disembarkation	0.0119	75
20	Memories	0.0113	75	20	Health	0.0115	72

**Table 6:** Top 20 ranked keywords for C1 and C2.

No.	Keywords	TF-IDF Value	Occurrences	No.	Keywords	TF-IDF Value	Occurrences
1	Happy	0.0257	721	1	Banks	0.0272	410
2	Doctors	0.0210	529	2	Epidemic	0.0213	285
3	New Year	0.0207	519	3	Hope	0.0193	247
4	Epidemic	0.0199	492	4	Withdrawal	0.0163	196
5	Hope	0.0129	268	5	Depositors	0.0151	177
6	Lovely	0.0125	256	6	Supplies	0.0145	167
7	Go for it!	0.0111	219	7	Life	0.0144	165
8	City	0.0110	215	8	Unblocked	0.0140	159
9	Subdivision	0.0105	203	9	Deposit	0.0140	159
10	Expectations	0.0091	169	10	Subdivision	0.0137	154
11	Vaccines	0.0090	166	11	Henan	0.0129	142
12	Government	0.0089	163	12	Villages and Towns	0.0124	135
13	Virus	0.0085	154	13	China	0.0118	127
14	People	0.0085	154	14	Nucleic acid	0.0118	127
15	Life	0.0084	152	15	Country	0.0116	124
16	Well	0.0083	150	16	Happy	0.0109	114
17	Pudong	0.0083	148	17	Government	0.0107	112
18	Isolation	0.0083	148	18	Online	0.0104	108
19	China	0.0082	147	19	Recovery	0.0102	105
20	Ambulance	0.0082	147	20	News	0.0101	104

**Table 7:** Top 20 keywords in C3 ranking.

No.	Keywords	TF-IDF Value	Occurrences
1	Typhoon	0.0255	240
2	Nucleic acid	0.0229	207
3	Child	0.0185	155
4	Go for it!	0.0167	136
5	Expectations	0.0162	130
6	Feelings	0.0161	129
7	Spokesperson	0.0156	124
8	Maotai	0.0143	111
9	Brands	0.0141	109
10	Global	0.0140	108
11	China	0.0133	101
12	Hope	0.0130	98
13	Support	0.0128	96
14	Courts	0.0128	96
15	Elderly people	0.0122	90
16	City	0.0117	85
17	Judges	0.0117	85
18	Not bad	0.0115	84
19	Police	0.0107	76
20	Epidemic	0.0104	74

**Table 8:** Number of themes January 2019–September 2022.

Year	Quarterly	Number	Number of Topics
2020	1	A1	8
	2	A2	5
	3	A3	7
	4	A4	5
2021	1	B1	9
	2	B2	6
	3	B3	8
	4	B4	4
2022	1	C1	4
	2	C2	7
	3	C3	4

**Table 9:** A1 LDA theme model results.

Topic1 Words	Probability Distribution	Topic2 Words	Probability Distribution	Topic3 Words	Probability Distribution	Topic4 Words	Probability Distribution
Support	0.155	Supervisor	0.099	China	0.075	Take a stand	0.373
Stories	0.087	United States	0.048	Mickey	0.058	Reply	0.361
Police Station	0.074	Video	0.034	City	0.032	World	0.025
Police	0.027	Government	0.023	Travel	0.032	Heroes	0.017
Documentary	0.024	Industrial	0.022	Zhejiang	0.020	Happy	0.014
Topic5 Words	Probability Distribution	Topic6 Words	Probability Distribution	Topic7 Words	Probability Distribution	Topic8 Words	Probability Distribution
Hard work	0.060	Mouthpiece	0.080	Happy	0.058	Go for it!	0.081
Heroes	0.042	Beijing	0.043	Happiness	0.048	Excellent	0.045
Blessings	0.038	Hospital	0.031	First	0.037	Acting	0.041
Go Home	0.034	Wuhan	0.028	Bicycles	0.036	Hope	0.035
Doctors	0.025	Epidemic	0.025	Hahaha	0.024	Shanghainese	0.033

**Table 10:** A2 LDA theme model results.

Topic1 Words	Probability Distribution	Topic2 Words	Probability Distribution	Topic3 Words	Probability Distribution
Testing	0.038	Wages	0.056	Contract Renewal	0.029
Nanjing	0.033	Demolition and relocation	0.024	Revenue	0.025
Railroad	0.027	Private	0.020	Spring	0.023
Start of school	0.025	Night Market	0.016	Air Conditioning	0.021
Infection	0.016	House price	0.015	Transportation	0.017
Topic4 Words	Probability Distribution	Topic5 Words	Probability Distribution		
High Speed Rail	0.048	Good to see	0.063		
Hours	0.035	Go for it!	0.050		
Xi’an	0.032	Awesome	0.041		
Chengdu	0.029	First	0.032		
Guangzhou	0.028	Summer Tournament	0.031		

**Table 11:** A3 LDA theme model results.

Topic1 Words	Probability Distribution	Topic2 Words	Probability Distribution	Topic3 Words	Probability Distribution	Topic4 Words	Probability Distribution
Rescue	0.078	Games	0.129	Expectations	0.135	Specimens	0.138
Lovely	0.072	Itinerary	0.060	Congratulations	0.102	Illegal	0.052
Animals	0.066	Specialties	0.041	Juvenile	0.070	Institution	0.050
Protection	0.056	Video	0.020	Good to see	0.065	Country	0.039
Wild	0.045	Strength	0.019	The future is promising	0.053	Hospital	0.032
Topic5 Words	Probability Distribution	Topic6 Words	Probability Distribution	Topic7 Words	Probability Distribution		
Queue	0.045	Illegal	0.052	Happy	0.068		
Department	0.042	Registration	0.052	Birthday	0.053		
Follow Super Talk	0.038	Official	0.036	Delicious	0.038		
Opportunities	0.035	Link	0.031	Value	0.024		
Works	0.026	Web	0.024	Sunshine Rose	0.022		

**Table 12:** A4 LDA theme model results.

Topic1 Words	Probability Distribution	Topic2 Words	Probability Distribution	Topic3 Words	Probability Distribution
Stage	0.052	Outerwear	0.075	Disney	0.050
Crossover	0.046	Hard work	0.058	Beautiful	0.039
Expectations	0.041	Go for it!	0.050	Live	0.016
Beautiful	0.032	So sweet	0.047	Guangzhou	0.016
Hope	0.030	Milk Tea	0.025	Beijing	0.016
Topic4 Words	Probability Distribution	Topic5 Words	Probability Distribution	Topic6 Words	Probability Distribution
Happy	0.120	Long skirt	0.054		
New Year	0.114	Embroidery	0.054		
Good to see	0.082	Diamond jewelry	0.053		
Fireworks	0.028	Clothes	0.023		
Next year	0.027	Blue Diamond	0.018		

**Table 13:** B1 LDA theme model results.

Topic1 Words	Probability Distribution	Topic2 Words	Probability Distribution	Topic3 Words	Probability Distribution
Clock in	0.031	Go for it!	0.037	Hope	0.047
Freedom	0.031	Life	0.033	Country	0.044
Dressing	0.024	Injury	0.026	Local	0.022
Opportunities	0.022	Friends	0.019	Chengdu	0.020
Photo	0.019	Mood	0.019	Guangzhou	0.014
Topic4 Words	Probability Distribution	Topic5 Words	Probability Distribution	Topic6 Words	Probability Distribution
Star	0.029	Legal	0.110	Hard work	0.049
Registration	0.017	Fairness	0.090	Borrowing	0.033
The Truth	0.013	Fairness	0.060	Paying off debts	0.029
Illegal	0.013	Support	0.025	Return	0.028
Facts	0.012	According to the law	0.015	Right and proper	0.028
Topic7 Words	Probability Distribution	Topic8 Words	Probability Distribution	Topic9 Words	Probability Distribution
Expectations	0.052	Opinion	0.059	Beautiful	0.035
Large film	0.041	Courts	0.026	Video	0.029
Flavor	0.037	Live Streaming	0.023	Fashion	0.015
Suits	0.037	Legal	0.023	Design	0.014
So handsome	0.036	Disinformation	0.018	Absent	0.013

**Table 14:** B2 LDA theme model results.

Topic1 Words	Probability Distribution	Topic2 Words	Probability Distribution	Topic3 Words	Probability Distribution
Good to see	0.070	Movies	0.050	Meet and greet	0.017
Hope	0.036	Lake Changjin	0.042	Chongqing	0.015
First	0.035	Live Streaming	0.021	City	0.015
Time	0.023	Screening	0.017	Changsha	0.013
Tomorrow	0.018	Support	0.017	Itinerary	0.013
Topic4 Words	Probability Distribution	Topic5 Words	Probability Distribution	Topic6 Words	Probability Distribution
Birthday	0.064	China	0.036	Expectations	0.128
May Day	0.025	Beijing	0.032	Stage	0.025
Disney	0.019	Jobs	0.018	Clock in	0.020
Graduation	0.018	Hangzhou	0.013	Opportunities	0.019
June 1	0.018	Grab a Ticket	0.012	Live	0.018

**Table 15:** B3 LDA theme model results.

Topic1 Words	Probability Distribution	Topic2 Words	Probability Distribution	Topic3 Words	Probability Distribution	Topic4 Words	Probability Distribution
Wonderful	0.043	Developers	0.028	Envy	0.057	Little brother	0.056
Video	0.038	House	0.027	Hope	0.035	Takeaway	0.050
Kudos	0.037	China	0.027	City	0.032	Attitude	0.026
Photo	0.023	Air Conditioning	0.021	Beijing	0.026	Elderly people	0.024
Live Streaming	0.021	Policy	0.017	Arrangement	0.022	Women	0.023
Topic5 Words	Probability Distribution	Topic6 Words	Probability Distribution	Topic7 Words	Probability Distribution	Topic8 Words	Probability Distribution
Support	0.039	Child	0.073	Miss	0.035	Hard work	0.038
Time	0.035	Marriage	0.050	Atmosphere	0.033	Local	0.033
Positive Energy	0.034	House price	0.031	Years	0.023	Microblog	0.025
Competition	0.024	Second child	0.028	Effort	0.022	Epidemic	0.024
After reading	0.020	Buy a house	0.017	Brands	0.016	Thank you	0.019

**Table 16:** B4 LDA theme model results.

Topic1 Words	Probability Distribution	Topic2 Words	Probability Distribution	Topic3 Words	Probability Distribution	Topic4 Words	Probability Distribution
Disney	0.045	Go for it!	0.030	Happy	0.072	Beijing	0.027
Atmosphere	0.030	China	0.026	New Year	0.057	City	0.022
Epidemic	0.022	Hard work	0.023	Exhibition	0.031	Happiness	0.021
Halloween	0.019	Expectations	0.017	Fireworks	0.020	Chengdu	0.021
Nucleic acid	0.018	First	0.015	Disembarkation	0.018	Local	0.017

**Table 17:** C1 LDA theme model results.

Topic1 Words	Probability Distribution	Topic2 Words	Probability Distribution	Topic3 Words	Probability Distribution	Topic4 Words	Probability Distribution
Epidemic	0.041	Local	0.018	Doctors	0.025	Happy	0.057
Isolation	0.016	Changsha	0.016	Vaccines	0.015	New Year	0.042
Nucleic acid	0.016	Country	0.014	Subdivision	0.013	New Year’s Eve	0.015
Hard work	0.014	Management	0.009	Ambulance	0.009	Unblocked	0.013
People	0.013	Beijing	0.009	Seal the City	0.008	Zeroing	0.012

**Table 18:** C2 LDA theme model results.

Topic1 Words	Probability Distribution	Topic2 Words	Probability Distribution	Topic3 Words	Probability Distribution	Topic4 Words	Probability Distribution
Banks	0.025	Northeast	0.358	Epidemic	0.045	Unblocked	0.024
Deposit	0.016	South	0.313	Resident Council	0.025	Government	0.018
Withdrawal	0.015	Wages	0.048	Local	0.017	Nucleic acid	0.016
Depositors	0.015	Economy	0.045	Jobs	0.014	Elderly people	0.013
Recovery	0.014	City	0.044	Home	0.013	Street	0.013
Topic5 Words	Probability Distribution	Topic6 Words	Probability Distribution	Topic7 Words	Probability Distribution		
Supplies	0.021	Level	0.130	Prices of goods	0.210		
Subdivision	0.019	Ordinary people	0.035	China	0.025		
Social	0.015	Hard-earned money	0.016	Zeroing	0.014		
Beijing	0.014	Consumption	0.015	Data	0.011		
People	0.012	Company	0.012	Go to work	0.011		

**Table 19:** C3 LDA theme model results.

Topic1 Words	Probability Distribution	Topic2 Words	Probability Distribution	Topic3 Words	Probability Distribution	Topic4 Words	Probability Distribution
Typhoon	0.045	United States	0.035	Rumors	0.020	Disinformation	0.033
Nucleic acid	0.033	City	0.026	Courts	0.014	Support	0.033
Refute the rumor	0.018	China	0.019	Network	0.013	Civilian Police	0.016
Time	0.012	Hangzhou	0.018	Scouting	0.012	First	0.013
Local	0.011	Global	0.016	Epidemic	0.010	Recommendation	0.013

**Table 20:** Normalized sentiment score.

Year	Quarterly	Scoring Normalization
2020	1	0.019050095
2020	2	0.0061449
2020	3	0.067988614
2020	4	0.025622107
2021	1	0.011496726
2021	2	0.024320776
2021	3	0.059270272
2021	4	0.021147083
2022	1	0.006401534
2022	2	−0.006365506
2022	3	−0.014056512

**Figure A1:**
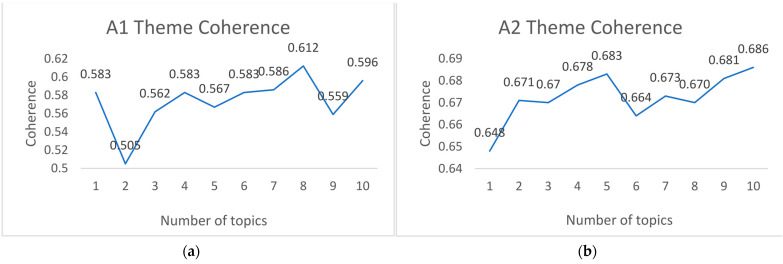

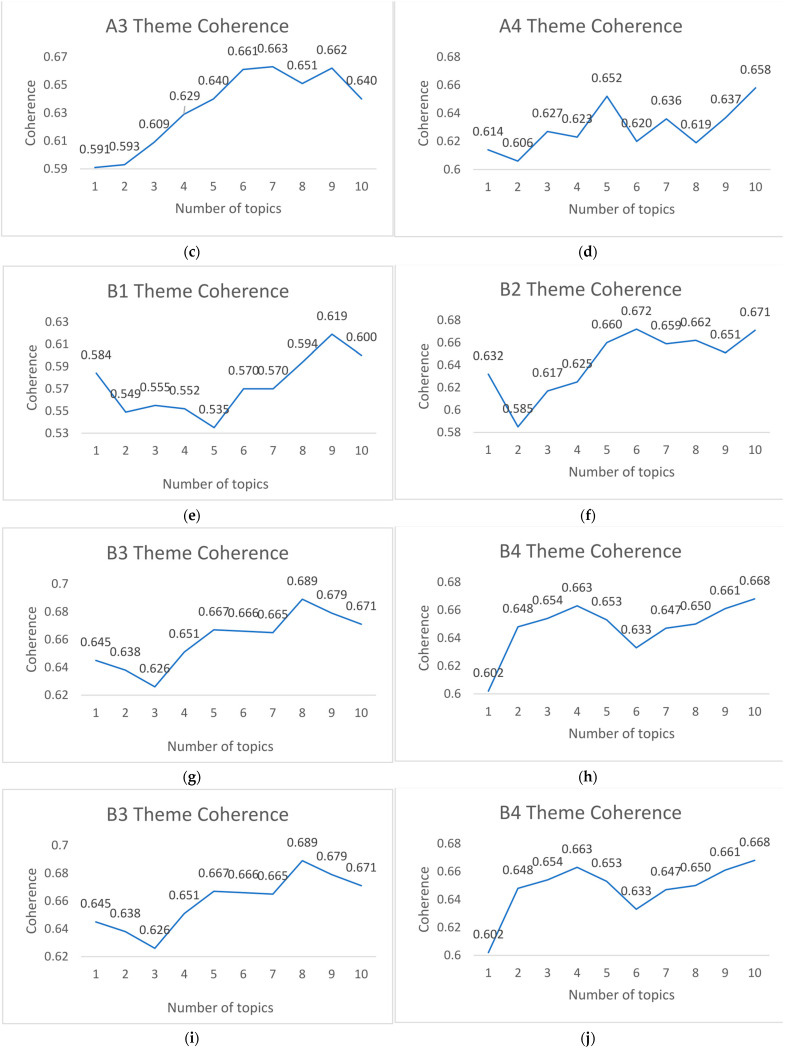

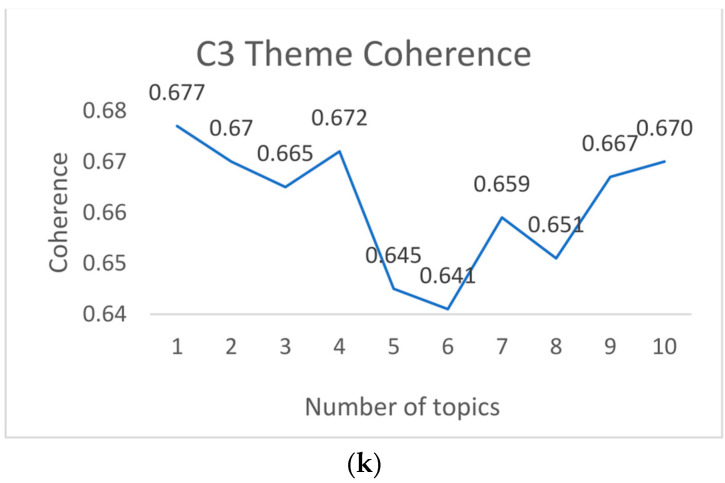
(**a**)A1 LDA Theme—Coherence; (**b**) A2 LDA Theme—Coherence; (**c**) A3 LDA Theme—Coherence; (**d**) A4 LDA Theme—Coherence; (**e**) B1 LDA Theme—Coherence; (**f**) B2 LDA Theme—Coherence; (**g**) B3 LDA Theme—Coherence; (**h**) B4 LDA Theme—Coherence; (**i**) C1 LDA Theme—Coherence; (**j**) C2 LDA Theme—Coherence; (**k**) C3 LDA Theme—Coherence.

## Data Availability

The experiment data used to support the findings of this study are included in the article.

## References

[1] White E.V., Gatersleben B. (2011). Greenery on Residential Buildings: Does It Affect Preferences and Perceptions of Beauty?. J. Environ. Psychol..

[2] Rohleder N. (2019). Stress and Inflammation—The Need to Address the Gap in the Transition between Acute and Chronic Stress Effects. Psychoneuroendocrinology.

[3] Dai T., Zhuang T., Yan J., Zhang T. (2018). From Landscape to Mindscape: Spatial Narration of Touristic Amsterdam. Sustainability.

[4] Cinelli M., Quattrociocchi W., Galeazzi A., Valensise C.M., Brugnoli E., Schmidt A.L., Zola P., Zollo F., Scala A. (2020). The COVID-19 Social Media Infodemic. Sci. Rep..

[5] Kemp S. Digital 2021: Global overview report. DataReportal – Global Digital Insights. https://datareportal.com/reports/digital-2021-global-overview-report.

[6] Data Home-Weibo Data Center-Sina Weibo Weibo.com. https://data.weibo.com/datacenter/.

[7] Bujnowska-Fedak M.M., Waligóra J., Mastalerz-Migas A. (2019). The Internet as a Source of Health Information and Services. Advancements and Innovations in Health Sciences.

[8] Laaksonen P., Laaksonen M., Borisov P., Halkoaho J. (2006). Measuring Image of a City: A Qualitative Approach with Case Example. Place Brand..

[9] Kai B., Anzhou Z. (2001). Studies on Convergence and Divergence of City Image and Destination Image. Prog. Geogr..

[10] Chapman E.H., Lynch K. (1962). The Image of the City. J. Aesthet. Art Crit..

[11] Hunt J.D. (1975). Image as a Factor in Tourism Development. J. Travel Res..

[12] Chon K.-S. (1990). The Role of Destination Image in Tourism: A Review and Discussion. Rev. Tour..

[13] Baloglu S., McCleary K.W. (1999). A Model of Destination Image Formation. Ann. Tour. Res..

[14] Hong S.-K., Kim J.-H., Jang H., Lee S. (2006). The Roles of Categorization, Affective Image and Constraints on Destination Choice: An Application of the NMNL Model. Tour. Manag..

[15] Chen X., Li J., Han W., Liu S. (2021). Urban Tourism Destination Image Perception Based on LDA Integrating Social Network and Emotion Analysis: The Example of Wuhan. Sustainability.

[16] Foot J.M. (1980). From Boomtown to Bribesville; the Images of the City. Urban Hist.

[17] Al-ghamdi S.A., Al-Harigi F. (2015). Rethinking Image of the City in the Information Age. Procedia Comput. Sci..

[18] Martí P., Serrano-Estrada L., Nolasco-Cirugeda A. (2019). Social Media Data: Challenges, Opportunities and Limitations in Urban Studies. Comput. Environ. Urban Syst..

[19] Priporas C.-V., Stylos N., Kamenidou I. (2020). City Image, City Brand Personality and Generation Z Residents’ Life Satisfaction under Economic Crisis: Predictors of City-Related Social Media Engagement. J. Bus. Res..

[20] Lin M.S., Liang Y., Xue J.X., Pan B., Schroeder A. (2021). Destination Image through Social Media Analytics and Survey Method. Int. J. Contemp. Hosp. Manag..

[21] Pan X., Rasouli S., Timmermans H. (2021). Investigating Tourist Destination Choice: Effect of Destination Image from Social Network Members. Tour. Manag..

[22] Huang J., Obracht-Prondzynska H., Kamrowska-Zaluska D., Sun Y., Li L. (2021). The Image of the City on Social Media: A Comparative Study Using “Big Data” and “Small Data” Methods in the Tri-City Region in Poland. Landsc. Urban Plan..

[23] Luque-Martínez T., Del Barrio-García S., Ibáñez-Zapata J.Á., Rodríguez Molina M.Á. (2007). Modeling a City’s Image: The Case of Granada. Cities.

[24] Nska A.-M., Michnik A., Polok J. (2019). A Systemic Approach to City Image Building. The Case of Katowice City. Sustainability.

[25] Kourtit K., Neuts B., Nijkamp P., Wahlström M.H. (2021). A Structural Equation Model for Place-Based City Love: An Application to Swedish Cities. Int. Reg. Sci. Rev..

[26] Bhavaraju T., Beyney S.K., Nicholson C. (2019). Quantitative Analysis of Social Media Sensitivity to Natural Disasters. Int. J. Disaster Risk Reduct..

[27] Kumar A., Singh J.P., Dwivedi Y.K., Rana N.P. (2020). A Deep Multi-Modal Neural Network for Informative Twitter Content Classification during Emergencies. Ann. Oper. Res..

[28] Ray A., Bala P.K. (2020). Social Media for Improved Process Management in Organizations during Disasters. Knowl. Proc. Manag..

[29] Special Expert Group for Control of the Epidemic of Novel Coronavirus Pneumonia of the Chinese Preventive Medicine Association (2020). An Update on the Epidemiological Characteristics of Novel Coronavirus Pneumonia (COVID-19). Zhonghua Liu Xing Bing Xue Za Zhi.

[30] Liu Q., Gao Y., Chen Y. (2014). Study on Disaster Information Management System Compatible with VGI and Crowdsourcing. Proceedings of the 2014 IEEE Workshop on Advanced Research and Technology in Industry Applications (WARTIA).

[31] Michael F., Goodchild J., Glennon A. (2010). Crowdsourcing Geographic Information for Disaster Response: A Research Frontier. Int. J. Digit. Earth.

[32] Chae J., Thom D., Jang Y., Kim S., Ertl T., Ebert D.S. (2014). Public Behavior Response Analysis in Disaster Events Utilizing Visual Analytics of Microblog Data. Comput. Graph..

[33] Steiger E., Resch B., Zipf A. (2016). Exploration of Spatiotemporal and Semantic Clusters of Twitter Data Using Unsupervised Neural Networks. Geogr. Inf. Syst..

[34] Miller H.J., Goodchild M.F. (2015). Data-Driven Geography. GeoJournal.

[35] Politis I., Georgiadis G., Kopsacheilis A. (2021). Capturing Twitter Negativity Pre-vs. Mid-COVID-19 Pandemic: An LDA Application on London Public Transport System. Sustainability.

[36] Dahal B., Kumar S.A.P., Li Z. (2019). Topic Modeling and Sentiment Analysis of Global Climate Change Tweets. Soc. Netw. Anal. Min..

[37] Ye X., Li S., Yang X., Qin C. (2016). Use of Social Media for the Detection and Analysis of Infectious Diseases in China. ISPRS Int. J. Geoinf..

[38] Ko J., Paek S., Park S. (2021). A News Big Data Analysis of Issues in Higher Education in Korea amid the COVID-19 Pandemic. Sustainability.

[39] Zong Q., Yang S., Chen Y., Shen H. (2015). Behavior of Social Media Users in Disaster Area under the Outburst Disasters: A Content Analysis and Longitudinal Study of Explosion in Tianjin 12(th) August 2015. Explos. Tianjin.

[40] Peng X., Bao Y., Huang Z. (2020). Perceiving Beijing’s “City Image” across Different Groups Based on Geotagged Social Media Data. IEEE Access.

[41] Zucco C., Calabrese B., Agapito G., Guzzi P.H., Cannataro M. (2020). Sentiment Analysis for Mining Texts and Social Networks Data:Methods and Tools. Interdiscip. Rev. Data Min. Knowl. Discov..

[42] Sankar H., Subramaniyaswamy V. (2017). Investigating Sentiment Analysis Using Machine Learning Approach. Proceedings of the 2017 International Conference on Intelligent Sustainable Systems (ICISS).

[43] Kwok S.W.H., Vadde S.K., Wang G. (2021). Tweet Topics and Sentiments Relating to COVID-19 Vaccination among Australian Twitter Users: Machine Learning Analysis. J. Med. Internet Res..

[44] Ahmed M.S., Aurpa T.T., Anwar M.M. (2021). Detecting Sentiment Dynamics and Clusters of Twitter Users for Trending Topics in COVID-19 Pandemic. PLoS ONE.

[45] Ridhwan K.M., Hargreaves C.A. (2021). Leveraging Twitter Data to Understand Public Sentiment for the COVID-19 Outbreak in Singapore. Int. J. Inf. Manag. Data Insights.

[46] Samuel J., Ali G.G.M.N., Rahman M.M., Esawi E., Samuel Y. (2020). COVID-19 Public Sentiment Insights and Machine Learning for Tweets Classification. Information.

[47] Gencoglu O., Gruber M. (2020). Causal Modeling of Twitter Activity during COVID-19. Computation.

[48] Bailón-Elvira J.C., Cobo M.J., Herrera-Viedma E., López-Herrera A.G. (2019). Latent Dirichlet Allocation (LDA) for Improving the Topic Modeling of the Official Bulletin of the Spanish State (BOE). Procedia Comput. Sci..

[49] Rashid J., Shah S.M.A., Irtaza A. (2019). Fuzzy Topic Modeling Approach for Text Mining over Short Text. Inf. Process. Manag..

[50] Pavlinek M., Podgorelec V. (2017). Text Classification Method Based on Self-Training and LDA Topic Models. Expert Syst. Appl..

[51] Newman D., Bonilla E.V., Buntine W. Improving Topic Coherence with Regularized Topic Models. Proceedings of the 25th Annual Conference on Neural Information Processing Systems.

[52] Stevens K., Kegelmeyer P., Andrzejewski D. Exploring Topic Coherence over Many Models and Many Topics. Proceedings of the 2012 Joint Conference on Empirical Methods in Natural Language Processing and Computational Natural Language Learning.

[53] Röder M., Both A., Hinneburg A. (2015). Exploring the Space of Topic Coherence Measures. Proceedings of the Eighth ACM International Conference on Web Search and Data Mining—WSDM’15, Shanghai, China, 2–6 February 2015.

[54] Yue A., Mao C., Chen L., Liu Z., Zhang C., Li Z. (2022). Detecting Changes in Perceptions towards Smart City on Chinese Social Media: A Text Mining and Sentiment Analysis. Buildings.

[55] Luo R., Xu J., Zhang Y., Zhang Z., Ren X., Sun X. (2019). A Toolkit for Multi-Domain Chinese Word Segmentation. arXiv.

[56] Pine B.J., Gilmore J.H. (1998). The Experience Economy: Work Is Theater and Every Business A Stage.

[57] Haihong E., Yingxi H., Haipeng P., Wen Z., Siqi X., Peiqing N. (2019). Theme and Sentiment Analysis Model of Public Opinion Dissemination Based on Generative Adversarial Network. Chaos Solitons Fractals.

